# Magnetic Ion Channel Activation (MICA)-Enabled Screening Assay: A Dynamic Platform for Remote Activation of Mechanosensitive Ion Channels

**DOI:** 10.3390/ijms24043364

**Published:** 2023-02-08

**Authors:** Afeesh Rajan Unnithan, Michael Rotherham, Hareklea Markides, Alicia J. El Haj

**Affiliations:** 1 Healthcare Technology Institute, Institute of Translational Medicine, University of Birmingham, Birmingham B15 2TH, UK; 2 Centre for Pharmaceutical Engineering Science, School of Pharmacy and Medical Sciences, Faculty of Lifesciences, University of Bradford, Bradford BD7 1DP, UK

**Keywords:** magnetic nanoparticles, mechanosensitive ion channels, mechano-transduction, calcium assay, drug screening, nanomedicine

## Abstract

This study reports results of a mechanical platform-based screening assay (MICA) to evaluate the remote activation of mechanosensitive ion channels. Here, we studied ERK pathway activation and the elevation in intracellular Ca^2+^ levels in response to the MICA application using the Luciferase assay and Fluo-8AM assay, respectively. Functionalised magnetic nanoparticles (MNPs) targeting membrane-bound integrins and mechanosensitive TREK1 ion channels were studied with HEK293 cell lines under MICA application. The study demonstrated that active targeting of mechanosensitive integrins via RGD (Arginylglycylaspartic acid) motifs or TREK1 (KCNK2, potassium channel subfamily K member 2) ion channels can stimulate the ERK pathway and intracellular calcium levels compared to non-MICA controls. This screening assay offers a powerful tool, which aligns with existing high-throughput drug screening platforms for use in the assessment of drugs that interact with ion channels and influence ion channel-modulated diseases.

## 1. Introduction

The potential of nanomedicine, particularly in the areas of drug delivery and therapeutics, has been rapidly increasing in recent years. Among those, magnetic nanoparticles (MNPs) have shown potential in biomedical applications spanning imaging, diagnosis, drug delivery, and cancer therapy [1,2,3]. Since MNPs can be controlled, detected, and manipulated by an external magnetic field, it enables the widespread opportunity for use in non-invasive in vivo and in vitro applications [4].

The Magnetic Ion Channel Activation (MICA) platform can provide a remote mechanical stimulus, which in turn can trigger the mechanosensitive ion channels (MS) of the targeted cells to initiate a series of cellular signalling pathways [5]. MICA provides a powerful tool for the remote actuation of cells with significant advantages over the conventionally available techniques, mainly nanoindentation, optical tweezers, atomic force microscope (AFM) cantilevers, etc. [6,7]. Nanoindentation, which is also known as depth sensing indentation, was used here, and the indentation load-depth-time (P-δ-t) profiles were recorded. However, optical tweezers were employed directly to impose pN forces at the length scale of the single cell. Optical tweezers were mainly optimised to 2D single-cell scenarios and less attractive for complex 3D multicellular tissues and organoids. Similarly, using AFM, the tip of the AFM cantilever can be conjugated with small molecules, including ligands and antibodies, which enables manipulation of membrane proteins at nanometer precision with picoNewton sensitivity. All these methods involve complex machinery and difficulty in handling samples. So, the notable advantage of the MICA application over these conventional methods is its ability for ‘remote access’ to manipulate cell signalling pathways [8]. This will open a plethora of possibilities in detecting, preventing, and treating ion channel-related cellular dysfunctions and diseases aligned to current drug screening platforms and 3D tissue culture systems such as organoids.

Stimulation of mechanosensitive ion channels (MS) is crucial for the basic development and the functioning of various types of cells [9]. Moreover, they also play a vital role in cell and tissue repair, regeneration, pathology, and signalling [10,11]. TREK1 is a two-pore domain potassium channel (K2P) expressed in many tissue types including smooth muscle and neural tissues [8]. TREK1 ion channels are mechanosensitive and sensitive to other stimuli, such as temperature, intracellular pH, and free fatty acids. TREK1 is the most studied background K2P channel [12] and it is differentially regulated by a variety of chemical and physical stimuli, which make TREK1 a very promising and challenging target for the treatment of several pathologies [13]. Due to their unique molecular structure and easily accessible placing, ion channels have been identified as potential targets for various external stimulants. These include drugs like fluoxetine, gabapentine, carbamazepine, and valproate, which have been reported to interact with TREK1 channels [14,15,16]. Remote manipulation of functionalized nanoparticles has also been shown to modulate ion channels, which in turn can alter the intracellular signalling pathways, leading to a cascade of biological processes downstream [5,17,18]. Surface-functionalised MNPs are chemically and physically stable and environmentally safe, which along with their biocompatible nature, makes them valuable for such applications [19].

Based on this, we aimed at developing a 96-well plate-based screening assay to study MICA-dependent mechanosensitive cell stimulations using luciferase assay [20] and Fluo-8AM calcium-binding dye [21]. The null hypothesis in this study is that the mechanosensitive ERK signalling pathway and Ca^2+^ flux is unaffected by MNP-mediated mechano-activation by MICA. To test this, we used MICA to trigger ERK pathway activation and cytosolic Ca^2+^ flux in the respective cells. Signalling activity was measured in a HEK293 ERK reporter cell line using a microplate reader luciferase assay to monitor ERK activation and Fluo-8AM to measure Ca^2+^ flux, respectively. The MNPs were functionalised with either RGD to target the RGD-binding motifs of integrins or specific antibodies to target the loop region of the mechanosensitive ion channel TREK1 [22] (TREK1-MNPs), after which the targets were subjected to mechano-stimulation through MICA.

## 2. Results

**Figure 1:**
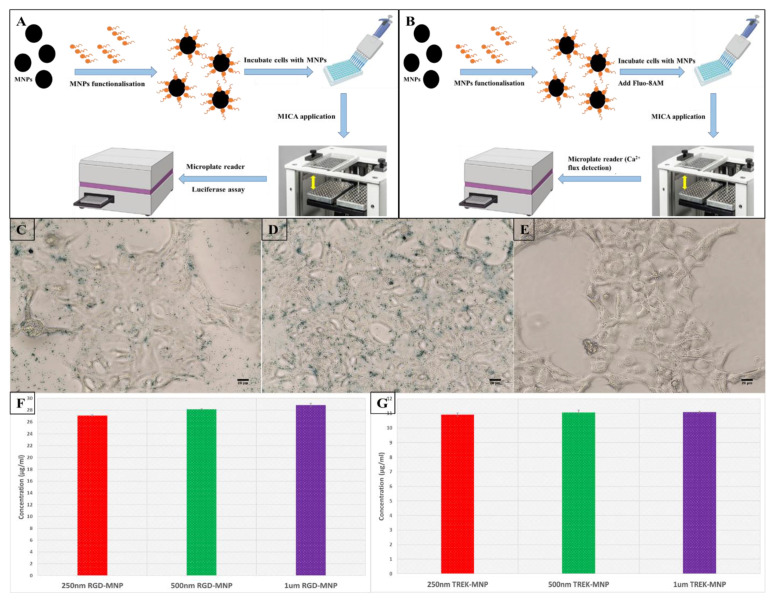
Graphic representation of experimental details of the application of MICA-based (**A**) Luciferase assay measurement procedure and (**B**) Fluo-8AM Ca^2+^ cytosolic Ca^2+^ levels’ detection procedure. Prussian blue-stained images of HEK293 cells labelled with (**C**) RGD-MNPs and (**D**) TREK1-MNPs compared to (**E**) non-labelled control cells. NanoOrange protein quantification results of (**F**) RGD-MNPs and (**G**) TREK1-MNPs.

MICA involves the application of an external permanent magnetic array oscillating at a frequency of 1 Hz, which establishes a magnetic gradient and applies a dynamic force to the functionalised MNPs bound to the respective receptor targets. This ultimately triggers the activation of downstream signalling through receptor mechano-transduction. The binding of functionalised 500 nm MNPs to HEK293 cells was visualised through Prussian blue staining, as seen in Figure 1C,D. The RGD-functionalised MNPs were mostly bound to the cell surface, and the TREK1-functionalised MNPs were seen to be internalised, as shown in Figure 1C,D, compared to the non-labelled cells in Figure 1E. As seen in Figure 1F, the NanoOrange protein quantification analysis of synthesised 20 µg/mL of stock RGD functionalised 250 nm, 500 nm, and 1 µm MNPs showed 27.05 ± 0.15 µg/mL, 28.12 ± 0.06 µg/mL, and 28.85 ± 0.31 µg/mL of RGD concentration, respectively. Similarly, in Figure 1G, the TREK1-Ab-functionalised 250 nm, 500 nm, and 1 µm MNPs showed 10.90 ± 0.12 µg/mL, 11.06 ± 0.15 µg/mL, and 11.08 ± 0.07 µg/mL, respectively, for a stock concentration of 10 µg/mL of TREK1 antibody. Hence, in both RGD-MNPs and TREK1-MNPs, a higher protein conjugation was observed, and these particles were introduced to cells for MICA-based cell stimulation. At first, the MICA platform was used to stimulate the MEK/ERK mitogen-activated protein kinase (MAPK) signalling cascade, which has a crucial influence on the expression of various proteins involved in the regulation of cell proliferation and differentiation [23].

**Figure 2:**
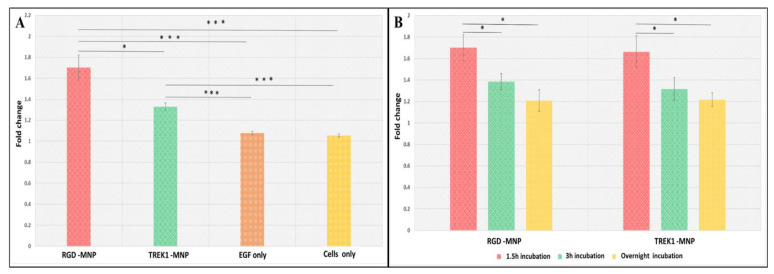
Luciferase assay exhibiting the (**A**) fold changes in the MEK/ERK pathway activation with RGD-MNPs and TREK1-MNPs, (**B**) effect of MNP incubation period (w/ and w/o MICA application, asterisks indicate significance in results using ANOVA test (* *p* < 0.05, *** *p* < 0.0005)).

The activation of the ERK pathway by MICA was examined using HEK-ERK luciferase reporter cells. The fold changes of ERK-dependent Luciferase activity increased in both RGD-MNP- and TREK1-MNP-treated cells after MICA treatment compared to the control groups as seen in Figure 2A. The results revealed that both RGD and anti-TREK1 targets showed significantly higher responses compared to the control groups. A similar positive response in the SRE reporter HEK293 cell line was obtained in response to treatment with EGF with or without a magnetic field. The data are shown as a ratio of magnetic to non-magnetic. Since the data are defined as fold change, the value of EGF is approximately one, as shown in Figure 2A.

### 2.1. Influence of MNP Incubation Period

We also examined the influence of the incubation period of the MNP with HEK cells (Figure 2B) on the ERK-dependent Luciferase activity and found that a higher incubation period results in lower luciferase expression with RGD- and anti-TREK1-functionalised nanoparticles possibly due to the internalization of the nanoparticles after increased exposure to the cells. As we are using magnetic field-assisted mechanical stimulation, a non-functionalised MNP sample cannot be used to replicate the mechanical or controlled temporal effects. An MNP control sample can cause non-specific binding and receptor clustering [24], which can evoke small levels of false-positive results in sensitive assays. In this study, we functionalised the MNPs with antibodies to target specific mechanosensitive regions of the ion channels to activate the cells in a controlled and targeted manner. From these results, it is evident that MICA, along with functionalised MNPs, can effectively target and remotely activate the ERK pathways in the targeted cells. The functional consequences of such ERK activation enhance the cellular and gene expression changes that promote cell proliferation, differentiation, and metabolism and, hence, are a crucial parameter for drug screening and cancer studies.

### 2.2. Impact of MNP Size in Fluo-8AM Response

**Figure 3:**
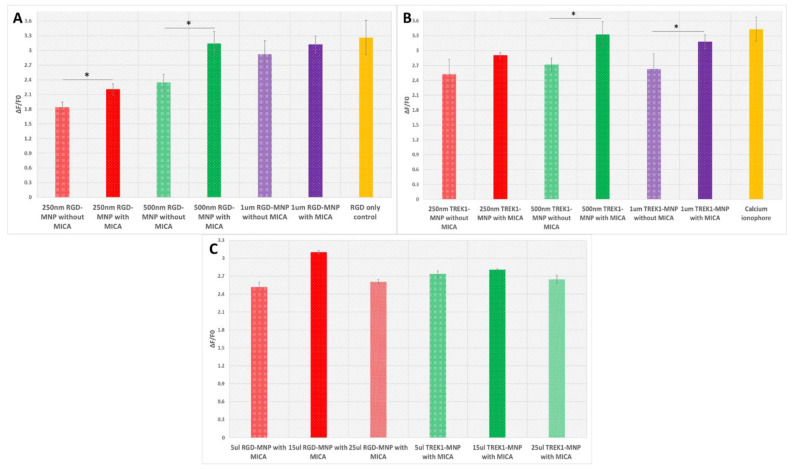
Size-dependent Fluo-8AM response with HEK293 cells labelled with (**A**) RGD-MNPs, (**B**) TREK1-MNPs, and (**C**) concentration-dependent Fluo-8AM response with RGD-MNPs and TREK1-MNPs after MICA application for 30 s (asterisks indicate significance in results using ANOVA test (* *p* < 0.05)).

**Figure 4:**
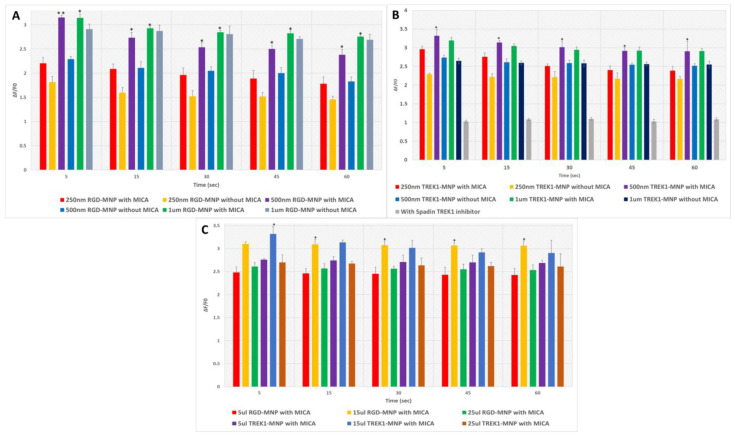
Time-dependent Fluo-8AM response with HEK293 cells labelled with (**A**) RGD-MNPs, (**B**) TREK1-MNPs, and (**C**) concentration-dependent Fluo-8AM response with time after MICA application for 30 s (asterisks indicate significance in results using ANOVA test (* *p* < 0.05, ** *p* < 0.01)).

The Fluo8AM, a fluorescent calcium-binding dye, will detect the intracellular cytosolic Ca2+ levels to MICA response. As seen in Figure 3A,B and Figure 4, both RGD- and anti-TREK1-functionalised MNPs showed a significant response in calcium signalling in HEK293 cells upon MICA application for 30 s. In the RGD-MNPs, 500 nm and 1 µm groups, a higher Fluo-8AM response was detected as compared to 250 nm particles (Figure 3A). As expected, the RGD only positive control group also showed a higher Fluo-8AM response by directly binding to the extracellular integrin receptors. The interactions of RGD-MNPs with extracellular integrin receptors depends on the morphological and physiological characteristics of MNPs. In particular, size is thought to play a key role in determining the efficiency of RGD-MNP-based cellular activation as it is targeted to an extracellular receptor [25]. The comparatively small 250 nm particles may be internalised faster compared to 500 nm and 1 µm RGD-MNPs, thus resulting in lower calcium responses compared to other groups.

Our results demonstrate that MICA-activated integrin–RGD-MNPs interactions can trigger cellular responses in HEK293 cells involved in calcium flux, which can offer further cellular stimulations.

The TREK1 mechanosensitive ion channels of HEK293 cells were targeted with TREK1-MNPs to the intracellular loop region and remotely activated through MICA (Figure 3B and Figure 4B). Activation of the TREK1 channel via MICA with 250 nm and 500 nm TREK1-MNPs demonstrated higher cytosolic Ca^2+^ levels compared to the MICA control group, and Ca^2+^ flux was blocked after addition of the TREK1 inhibitor Spadin (Figure 4B). As the TREK1-MNPs were targeted to the intracellular loop region, the internalization of the targeted particles is a crucial factor in determining the ion channel activation [5]. We observed a significantly higher response in both the 250 and 500 nm MNPs after 30 s of MICA activation compared to their control groups. Interestingly, with 1 µm TREK1-MNPs, the MICA group and control group did not possess any considerable variation in their Ca^2+^ response, clearly indicating the significance of size-dependent particle uptake in TREK1 activation. Calcium ionophore A23187 was introduced as a positive control to increase the Ca^2+^ concentration in the intracellular environment. Calcium ionophores are supposed to directly enable the passage of Ca^2+^ across the cell membrane and, hence, showed a higher response compared with MICA groups. TREK1 can also act as a gatekeeper for Ca^2+^ homeostasis; for instance, Yarishkin et al. demonstrated that pharmacological activation of TREK1 using ML-402 initiated increased intracellular Ca^2+^ [26]. Furthermore, TREK1 can facilitate the TRP channel-dependent Ca^2+^ influx, and TREK1-dependent hyperpolarisations can stimulate Ca^2+^ entry via tonically activated TRP-like channels [26,27,28]. Our data show the optimisation of the force on the receptor with increased particle size with the need for internalization. The preliminary results suggest that our MICA-based screening system with HEK293 cells is a powerful tool for developing a reliable model for drug screening and disease modelling and is a reliable platform to study metabolic diseases.

### 2.3. Effect of MNP Concentration in Fluo-8AM Response

We also evaluated the MNP concentration-based integrin and TREK1 channel remote activation with MICA. It was interesting to find that in HEK293 cell lines, 15 µL of MNPs solution (1 mg/mL stock) per well of the 96-well plates showed a higher cytosolic Ca^2+^ level compared to 5 µL and 25 µL of MNPs, as evidenced in Figure 3C, indicating that particle concentration also plays a crucial role in MICA-based ion channel activation. Regarding the 5 µL of MNPs, the concentration of particles may not be enough to achieve the threshold to activate the MS. At the same time, higher particle concentration can cause a blocking effect and even create a deteriorating effect on the integrity of cell membranes [29], which might cause the low cytosolic Ca^2+^ levels. So, considering all these facts, it is clear that we were successful in obtaining the optimum concentration required for the 96-well plate-based MICA-dependent Ca^2+^ activation.

## 3. Materials and Methods

Commercially available 250 nm and 500 nm MNPs (Nanomag from Micromod, Germany) and 1 µm MNPs (SiMAG, Chemicell, Germany) were functionalised with TREK1 antibody (Almone labs, APC-047, Israel) and RGD (Arg-Gly-Asp, A8052, Sigma Aldrich) as described elsewhere [8]. Peptide sequences with arginine-glycine-aspartic acid (RGD) motifs show a strong affinity for integrins, particularly αvβ3 [30,31]. These RGD peptide-functionalised MNPs (RGD-MNP) were used to target and activate the external integrin-dependent MS. We also initiated the optimisation of the protocol, assessing the influence of the size of the nanoparticles (250 nm, 500 nm, and 1 µm) and the concentration of targeted coating on the MICA-dependent remote activation of MS. The particles were functionalised with 1 µL DOTAP (N-[1-(2,3-dioleoyloxy)propyl]-N,N,N-trimethylammonium chloride, 1 µg/mL) to both promote cellular uptake and to prevent particle aggregation [22,32]. A custom-made MICA bioreactor (MICA Biosystems, West Midlands, UK, http://micabiosystems.com, accessed on 23 November 2022) kept inside an incubator (37 °C, 5%CO_2_) was used to magnetically stimulate the cells (Figure 1A). Control groups (without MICA and no MNP or MNP alone) were also kept at similar incubation conditions. The SRE reporter-HEK293 cell line (BPS biosciences, San Diego, CA, USA) was cultured in a growth medium (MEM medium supplemented with 10% FBS, 1% non-essential amino acids, 1 mM sodium pyruvate, 1% Penicillin/Streptomycin, and 400 µg/mL Geneticin). Calcium flux plays a vital role in all cellular activities, and hence, there is a growing demand for developing high-throughput measurements of Ca^2+^ using microplate readers for biopharmaceutical screening and drug development [33]. Moreover, calcium channels regulate the intracellular Ca^2+^ concentration playing a crucial role in cell proliferation, growth, and other necessary cellular functions. Interestingly, cytosolic Ca^2+^ level changes are very dynamic and therefore create challenges in high-throughput measurements. Hence, the MICA-dependent ERK pathway activation assessments in SRE reporter-HEK293 cell lines using the Luciferase assay and the MICA-dependent Fluo-8AM studies with HEK293 cells were done as described in Figure 1A,B, and the detailed protocol is given in the Supporting Information (SI). All fluorescence and luminescence measurements were recorded by a Spark 10 M multimode microplate reader (TECAN, Männedorf, Switzerland, 96-well plates (Thermo Fisher Scientific-Nunclon 96 Flat Transparent, Massachusetts, US) Fluo8AM for 30 s) at room temperature [34,35]. The Zeta potential evaluation before and after MNP functionalisation is given in the SI. Immunostaining, Western blotting, and rt-PCR were performed to confirm the expression of TREK1 in HEK293 cells, as shown in the Supporting Information (Supporting Information Figures S1 and S2).

## 4. Conclusions

In conclusion, we successfully developed a 96-well plate-based screening assay implementing MICA technology. Our preliminary results clearly state that the integrin-dependent MS and TREK1 channels were remotely manipulated successfully through functionalised MNPs using an external magnetic field (MICA), resulting in higher cytosolic Ca^2+^ levels and ERK pathway activation. Moreover, we also proved the importance of MNP size and concentration in determining the efficiency of the MICA screening assay. Both 500 nm RGD-MNPs and TREK1-MNPs with MICA showed a higher response compared to other MNPs. It is evident from our studies that MICA-based remote activation of mechanosensitive ion channels can be used as a powerful high-throughput tool for investigating the interaction between ion channels and the cell signalling in real time for various biopharmaceutical and drug screening applications.

### Statistical Testing

All experiments were performed in triplicates unless otherwise indicated. Data were analysed using the Origin statistical software from OriginLab Corporation, and data were presented as mean ± standard deviation SD (n = 3). The Student’s *t*-test or one-way ANOVA test was performed for comparison between two groups or among multiple groups, respectively, and statistically significant differences at the 95% confidence level were marked with * for *p* < 0.05, ** for *p* < 0.01, and *** for *p* < 0.001.

## Data Availability

Not applicable.

## Supplementary Materials

The following supporting information can be downloaded at: https://www.mdpi.com/article/10.3390/ijms24043364/s1. References [36,37,38,39] are cited in the supplementary materials.
