# Supplementary material for: Magnetic Ion Channel Activation (MICA)-Enabled Screening Assay: A Dynamic Platform for Remote Activation of Mechanosensitive Ion Channels
Source: Int J Mol Sci. 2023 Feb 8;24(4):3364. doi: 10.3390/ijms24043364 (PMC9962865; doi:10.3390/ijms24043364)
Supplement: Supplementary file 1 [file ijms-24-03364-s001.zip › ijms-2087355-supplementary.pdf]

Supporting Information

Magnetic Ion Channel Activation (MICA) Enabled Screening Assay: A Unique Dynamic Platform for Remote Activation of Mechanosensitive Ion-channels

Afeesh Rajan Unnithan[1], Michael Rotherham[1], Hareklea Markides[1], Alicia J. El Haj[1]

[1]Healthcare Technology Institute, Institute of Translational Medicine, University of Birmingham, Birmingham, B15 2TH, UK

[2]Centre for Pharmaceutical Engineering Science, School of Pharmacy and Medical Sciences, Faculty of Lifesciences, University of Bradford

Immunocytochemistry

The SRE Reporter – HEK293 cells were fixed with 10% paraformaldehyde for 10 minutes, permeabilised with 0.1% Triton™ X-100 for 10 minutes, blocked with 1% BSA for 1 hour and labelled with the anti-TREK-1 (Alomone Labs, #APC-047, 1:100) for 4 hours at room temperature. After washing the secondary antibody (Goat Anti-Rabbit IgG (FITC) (ab6717), 1:500) was added and incubated for 1 h at room temperature. The cells were washed and nuclei were stained with DAPI for 15min then washed and imaged under fluorescence microscope.

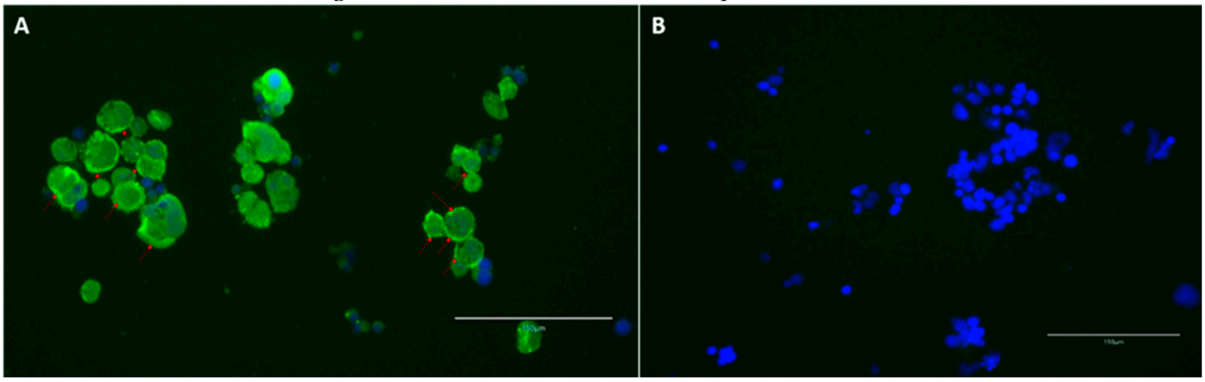

Figure S1. Immunocytochemistry imaging of SRE Reporter – HEK293 cell lines with antibodies against TREK-1 showing the presence of TREK-1 channels on the cell membrane (Red arrows) (A) and control cell sample without secondary antibody (B) (scale bar represents 150µm).

Western blotting

Western blotting was performed to confirm the presence of TREK1 on the HEK293 cells. Cells were washed with PBS then lysed in RIPA buffer containing protease and phosphatase inhibitor mixes (Sigma) for 15mins. The cell lysate was collected and transferred into Eppendorf tubes, clarified and the total protein was quantified using a µBCA assay (Fisher). For PAGE, 20µg of protein was mixed with LDS sample buffer and Bolt-sample reducing agent (Invitrogen). Samples were then heated for 10mins at 70°C then briefly centrifuged before loading onto Bis-Tris gels (Invitrogen) for PAGE. Proteins were transferred to Nitrocellulose membrane for 1h at 10v in a mini-blot module (Invitrogen). The membrane was blocked with 5% milk (Bio-rad) in TBS-T buffer (Sigma) for 1h. The membrane was then incubated with Anti-K2P2.1 (TREK-1) (Alomone labs) diluted 1:200 in 5% Milk in TBS-T overnight at room temperature under constant mixing. The membrane was washed 3x with TBS-T before incubation with Anti-rabbit-HRP (1:1000) (Abcam) for 1h at room temp. The membrane was washed 5x in TBS-T and developed using a West Pico Plus chemiluminescent kit (ThermoScientific). Image capture was performed using an iBright 1500 (Invitrogen).

Reverse transcription-PCR

Cells were washed with PBS then Total RNA was extracted using an RNA extraction kit (Bioline) according to the manufacturer's instructions. Reverse transcription was performed on 1μg RNA using a high capacity reverse transcription kit (Applied Biosystems). PCR reaction mixes were prepared by addition of diluted cDNA to PCR master mix (Applied Biosystems) and commercially available primers for KCNK2 (Qiagen). Thermocycling was performed on an AriaMx qPCR system (Agilent). PCR products were resolved on a 2% Agarose gel and imaged using an E-gel powersnap electrophoresis device (Invitrogen).

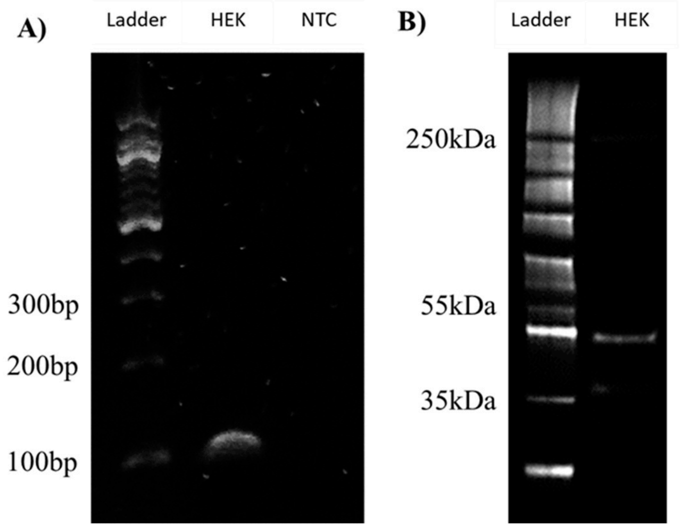

Figure S2: TREK1 is expressed at gene and protein level in HEK-ERK reporter cells. PCR was used to confirm TREK1 gene expression (A), NTC (non-template control). Western blot was also used to confirm TREK1 protein expression (B).

MICA based Luciferase assay procedure

The SRE reporter-HEK293 cell line (BPS biosciences, USA) was cultured in Growth medium 1B (MEM medium supplemented with 10% FBS, 1% non-essential amino acids, 1mM Na pyruvate, 1% Penicillin/Streptomycin and 400μg/ml Geneticin). For the experiments the cells were seeded to 96 well plates with a cell density of 3×10^4 /well and cultured under ambient cell culture conditions. After 24h the media was replaced with a serum free media. After 48h of culture the 15μl (1mg/ml stock) of RGD-MNP, RGD alone control (20μg/ml, positive control) and TREK1-MNPs with 1 μl DOTAP (1 μg/ml) to enhance permeabilisation [22, 36] and EGF (10ng/ml, positive control) were introduced to the respective wells and incubated for 1.5h. In case of incubation dependent studies the MNPs were incubated for 1.5h, 3h and overnight. After incubation, the particles were removed and washed with PBS to remove the residue particles and serum free media was introduced to each wells. Then the 96 well plates were introduced to the MICA platform and magnetically stimulated for 1h inside the incubator. After 1h of MICA application, the plates were incubated for 4h under the same ambient cell culture conditions and the Luciferase reagent was introduced thereafter. After thawing Luciferase reagent buffer in a room temperature water bath the luciferase assay working solution was prepared by diluting Luciferase reagent substrate into Luciferase reagent buffer at a 1:100 ratio and mix well. The 96 well plate containing the functionalised MNP tagged HEK 293 cells were removed from incubator and added an equal volume of luciferase assay working solution to the culture medium in each well. After gently rocking the plates for ≥15 minutes at room temperature, the respective luminescence was measured from each well using the microplate reader [37].

NanoOrange® protein quantification assay (ThermoFisher Scientific) is carried out according to the manufacturer's protocol. In short, 1X protein quantitation diluent was prepared by mixing the concentrated NanoOrange® protein quantitation diluent and diluted to 10-fold in distilled water. The NanoOrange® protein quantitation reagent was diluted to 500 times into the 1X protein quantitation diluent to prepare the 1X NanoOrange® reagent working solution. Finally the functionalised MNP solution is diluted in 1X NanoOrange® working solution to achieve a final volume of 250 μL in the 96 well plates. The functionalised MNP samples were added to the to the working solution and

incubated the samples at 96°C for 10 minutes and then cool down to room temperature. Later the fluorescence was measured by a Spark 10 M multimode microplate reader (TECAN, Switzerland) at room temperature with Ex (excitation) wavelength at approximately 485 nm and capturing the Em (emission) at approximately 590 nm [38, 39].

MICA depended Fluo-8AM assay procedure

For measuring the MICA based Fluo-8AM response, the cells (HEK293) were cultured in 96 well plates with a cell density of $3×10^4$ /well and were cultured under standard cell culture conditions as stated in the methods section previously. After 24h of culture the media was changed and serum free media was introduced. Then after 48h of culture the 15µl (1mg/ml stock) of RGD-MNP or TREK1-MNPs (250nm, 500nm and 1µm) were introduced to each wells. For concentration depended studies 5µl, 15µl or 25µl of MNPS (1mg/ml stock) were added to each wells. After 1.5h of incubation, the particles are removed and washed with PBS for removing the unbound particles and serum free media was added to each wells. 20µM Calcium Ionophore A23187 as a positive control for the assay was also added to the respective well. As a TREK1 inhibitor, 10 µM Spadin is also used. Later 5µM Fluo-8AM reagent (AAT Bioquest, 5mM stock) was introduced to each wells and incubated for 90min. Soon after the incubation, MICA stimulation was applied to the respective 96 well plates (Thermo Fisher Scientific-Nunclon 96 Flat Transparent) for 30s and immediately scanned by a Spark 10 M multimode microplate reader (TECAN, Switzerland) at room temperature at Ex (excitation) wavelength; 490nm and Em (emission) wavelength; 525nm.

Particle characterisation

The zeta potentials of MNPs before and after functionalisation was characterised with Zeta analyser (malvern zetasizer). As shown in the Table 1; 250nm, 500nm and 1µm MNPs showed an obvious decrease in zeta potential after conjugation with RGD and TREK1 antibody respectively. This indicate the presence of protein conjugation on the particle surface compared to the control MNPs which resulted in lower zeta compared to the control MNPs. It is evident from the Zeta potential values as given in Table 1. The zeta potential values were decreased after functionalisation with antibodies indicating the successful conjugation of the antibodies to the carboxylic groups on the surface of the MNPs.

Particle size	Zeta potential (mV)
250nmMNP	-12.7333± 0.20
250nmMNP-RGD	-11.4333 ± 0.05
250nmMNP-TREK	-10.5533 ± 1.00
500nmMNP	-19.6333 ± 0.23
500nmMNP-RGD	-14.5 ± 0.4
500nmMNP-TREK	-17.1333 ± 0.45
1µmMNP	-54.8667 ± 1.70
1µmMNP-RGD	-51.1667 ± 1.45
1µmMNP-TREK	-32.9 ± 1.75

Table S1. Zeta potential values of 250nm, 500nm and 1um MNPs before and after conjugation with RGD and TREK1 respectively. Values represent mean ± standard deviation.

1. [36] H.-Y.L. Anna-Maria Pappa, Walther Traberg-Christensen, Quentin Thiburce, Achilleas Savva, Aimie Pavia, Alberto Salleo, Susan Daniel, Rois´ ín M. Owens, Optical and Electronic Ion Channel Monitoring from Native Human Membranes, ACS Nano 14(10) (2020) 12538−12545.
2. [37] Q.A.Z. Wang, Y.X. Yang, X.L. Liang, LncRNA CTBP1-AS2 sponges miR-216a to upregulate PTEN and suppress endometrial cancer cell invasion and migration, J Ovarian Res 13(1) (2020).
3. [38] L.J. Jones, R.P. Haugland, V.L. Singer, Development and characterization of the NanoOrange (R) protein quantitation assay: A fluorescence-based assay of proteins in solution, Biotechniques 34(4) (2003) 850-+.
4. [39] S.L. Filbrun, J.D. Driskell, A fluorescence-based method to directly quantify antibodies immobilized on gold nanoparticles, Analyst 141(12) (2016) 3851-3857.
